# Ultrasound Assisted Catheter Directed Thrombolysis in the Management of a Right Atrial Thrombus: A New Weapon in the Armamentarium?

**DOI:** 10.1155/2016/4167397

**Published:** 2016-08-28

**Authors:** Mohamed Shokr, Ramanjit Kaur, Kevin Belgrave, Arshad Javed, Mahir Elder, Shaun Cardozo, Luis Afonso, Amir Kaki

**Affiliations:** ^1^Internal Medicine Department, Detroit Medical Center, Wayne State University, Detroit, MI, USA; ^2^Division of Cardiology, Detroit Medical Center, Wayne State University, Detroit, MI, USA

## Abstract

Catheter related thrombosis (CRT) is a commonly encountered entity fraught with substantial risk for mortality secondary to various complications including pulmonary embolism (PE), tricuspid regurgitation, endocarditis, right sided heart failure, and cardiogenic and septic shock. CRT carries a mortality rate of 18% in hemodialysis patients and more than 40% in nonhemodialysis patients. Management strategies include systemic anticoagulation, systemic thrombolysis, surgical evacuation, and percutaneous retrieval with no established guidelines. Ultrasound assisted catheter directed thrombolysis emerges as promising modality with a relatively lower risk of hemorrhage compared to systemic thrombolysis. We report a case of a 75-year-old man with dialysis catheter related thrombosis without PE for which ultrasound assisted catheter directed thrombolysis was used successfully as an alternative therapy.

## 1. Background

Catheter-related thrombosis (CRT) is a known complication of central venous catheters (CVC) with a reported incidence of 5–62% in asymptomatic thrombosis and 28% for symptomatic thrombosis. CRT carries a mortality rate of 18% in hemodialysis patients and more than 40% in nonhemodialysis patients [1]. Treatment options include systemic anticoagulation, systemic thrombolysis, surgical evacuation, and percutaneous retrieval with no established guidelines. We present a case of CRT for which ultrasound assisted catheter directed thrombolysis with an EKOS catheter was used as an alternative therapy.

## 2. Case Presentation

**Figure 1:**
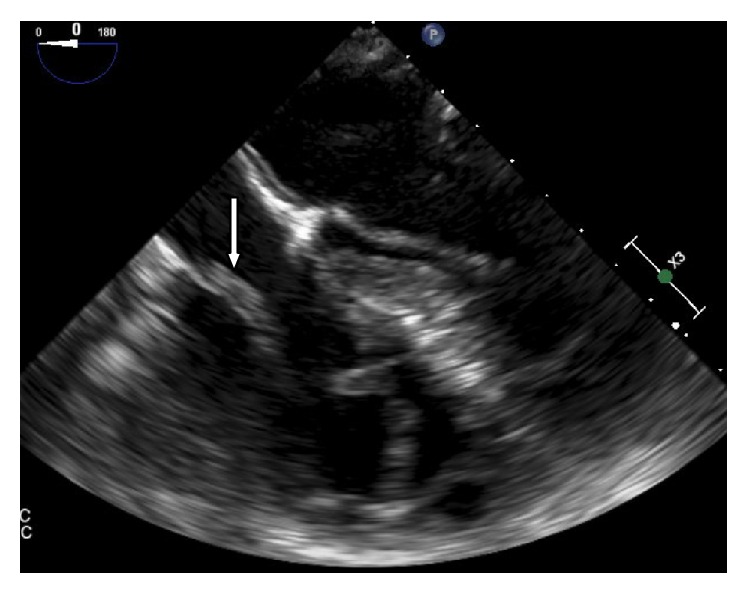
TEE image (apical four-chamber view) showing a serpiginous, elongated echodensity measuring 8.4 cm × 0.5 cm prolapsing into the tricuspid valve orifice deep into right ventricle; the attachment appears to be along the anterolateral right atrium.

**Figure 2:**
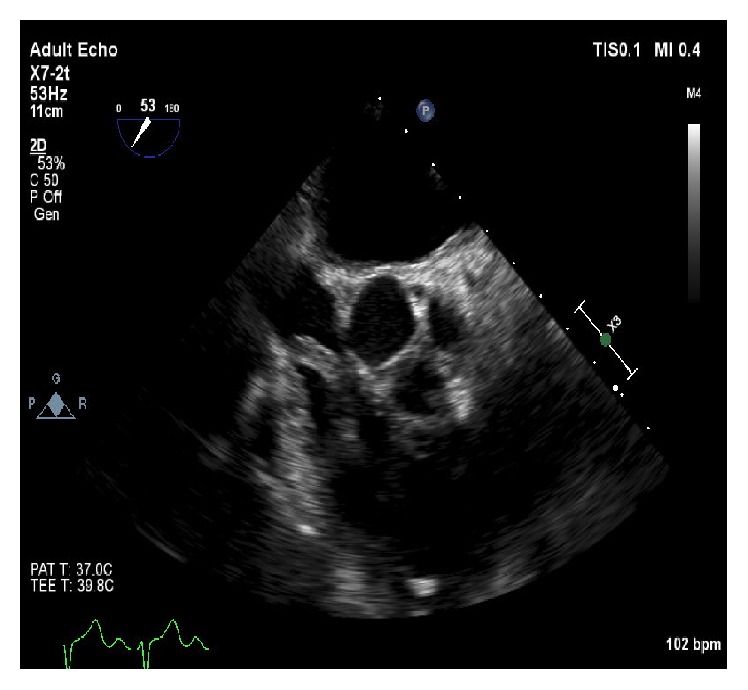
TEE image (short axis view at the aortic valve level) showing the thrombus.

**Figure 3:**
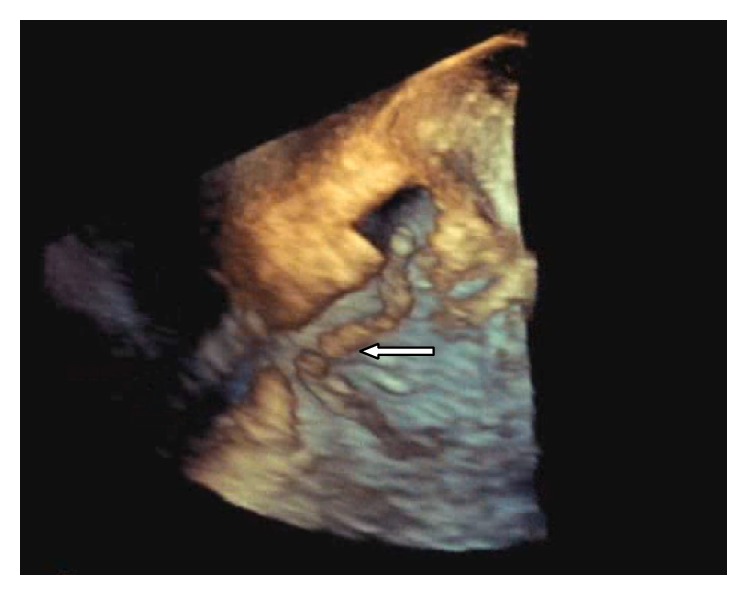
Three-dimensional view of the thrombus.

A 75-year-old gentleman with a past medical history of diabetes (DM), hypertension (HTN), prostate cancer status postprostatectomy, and end stage renal disease (ESRD) on hemodialysis was admitted from his dialysis center for an elective thrombectomy of his clotted left upper extremity arteriovenous graft (AVG). This finding had prompted insertion of a right subclavian permacath two weeks prior to admission. The patient was afebrile, with a blood pressure of 110/70, heart rate of 70 bpm, and an oxygen saturation of 97% on room air. On cardiovascular exam, he had normal S1 and S2 with no appreciated murmurs, rubs, gallops, jugular venous distension, or lower extremity edema. His basic lab panel was unremarkable and no leukocytosis was noted. He was originally scheduled for thrombectomy of the graft by vascular surgery but then was found to have asymptomatic Mobitz-I AV block on EKG which prompted cardiac evaluation. During this evaluation, a transthoracic echocardiogram (TTE) was significant for a prominent echodensity in the right atrium (RA). A subsequent transesophageal echocardiogram (TEE) demonstrated a serpiginous, highly mobile, elongated, oscillating echodensity measuring 8.4 cm × 0.5 cm prolapsing across the tricuspid valve orifice, probably consistent with thrombus [Figures 1, 2, and 3]. The thrombus was seen attached to the anterolateral aspect of the right atrium at what appeared to be the site of microtrauma ensuing from the tip of the deep seated permacath. Intravenous heparin was immediately started and the patient was boarded for direct thrombolysis via EKOS catheter which was inserted through a 7 French sheath into the left subclavian graft. A Wholey wire was advanced into the right ventricle (RV) and the EKOS catheter was placed through the dialysis line into the RA and through to the RV. An alteplase bolus of 5 mg was administered and was continued as a drip at a rate of 2 mg/hr for 10 hours.

**Figure 4:**
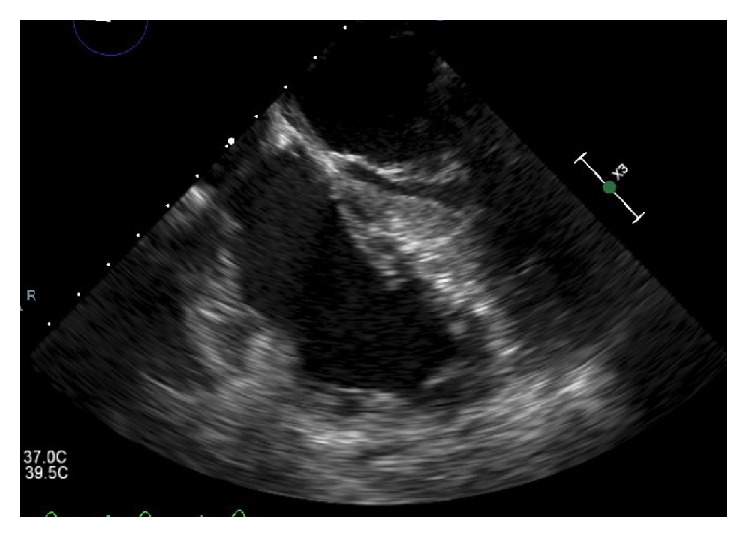
Post-EKOS TEE image (apical four-chamber view) showing resolution of previously noted serpiginous thrombus.

A follow-up TEE upon discontinuation of local thrombolytics showed resolution of the thrombus [Figure 4]. The patient was then started on warfarin. The hypercoagulability workup came back negative except for an elevated homocysteine level of 27 *μ*m/L (3.2–10). Our patient remained hemodynamically stable throughout his admission without suspicion of PE. Permacath was removed two weeks later.

## 3. Discussion and Review of Literature

CRT might be an underreported entity due to the asymptomatic nature and is usually detected after complications occur [2]. It is associated with different types of dialysis catheters and the incidence may vary according to venous site location or placement as well [1]. In one study of 55 patients with catheters placed into the RA, TEE revealed that 46% developed a thrombus within one week, in contrast to patients with catheters at the right atrial-superior vena caval junction who had no detected thrombi at week one or six. However, the National Kidney Foundation recommends right atrial positioning for better blood flow rates during dialysis [3].

CRT is associated with various complications including pulmonary embolism (PE) (4–6% of cases, with a three-month mortality rate of 16–29%), tricuspid regurgitation, partial obstruction of the tricuspid valve, endocarditis, right heart failure, electromechanical dissociation, cardiac arrest and cardiogenic or septic shock [1].

The postulated pathophysiologic mechanism leading to thrombosis includes activation of the coagulation cascade due to the repeated direct mechanical trauma to the right atrial wall caused by the catheter tip (most likely cause in our case as the thrombus was seen attached to the anterolateral aspect of the right atrium at what appeared to be the site of microtrauma ensuing from the tip of the deep seated permacath), exacerbated by altered fluid dynamics around the catheter in addition to intraluminal clot elongation and the increased thrombogenicity in hemodialysis patients [2].

In terms of the diagnostic modality, TEE has better sensitivity and specificity when compared to the TTE. In addition, 3D-echocardiography can provide higher resolution of the size, point of attachment, and mobility of the mass when compared to TTE [2].

Management strategies for CRT include anticoagulation, thrombolysis, surgical evacuation, and percutaneous retrieval, all combined with removal of the CVC after an initial period of anticoagulation; however there are no established guidelines or well characterized data on the incidence of various complications. Stavroulopoulos et al. concluded that no treatment choice was superior to the other in their meta-analysis of 71 cases of CRT in dialysis patients published as of December 2010 [1].

Among FDA approved thrombolytics for PE treatment, rtPA seems more favorable being fibrin-specific with a short half-life allowing surgical interventions in cases of thrombolytic failure [4]. Systemic thrombolysis accelerates thrombus lysis and pulmonary reperfusion and reduces pulmonary hypertension in cases of Type A thrombi with acute massive PE including those without hemodynamic compromise. Type A thrombi are highly mobile, free floating, emboli in transit from deep venous thrombosis, found in structurally normal atria and have a higher incidence of PE [1]. Systemic thrombolysis also lyses thrombi in different locations: lungs, RA, and the initial venous thrombus as well [4].

However thrombolysis is not as successful when it comes to type B thrombi which are those attached to the atrial wall and associated with structurally abnormal atrium or catheters. Stavroulopoulos et al. reported success in two patients among the eight who received thrombolytic therapy, one with pulmonary emboli and a large thrombus that extended from the right atrium to the right ventricle and to the left atrium via a patent foramen ovale and another after failed anticoagulation. This report further suggested that systemic thrombolysis use be reserved only for cases of failure of other approaches or those with concomitant massive PE [1].

Systemic thrombolysis carries a 22% risk of major hemorrhage including a 3% risk of intracranial hemorrhage as well as a high risk for fragmentation and distal embolization when used for large mobile thrombi leading to recurrent PE [5].

Surgical thrombectomy is one of the treatment options, usually under cardiopulmonary bypass. It is recommended in scenarios such as contraindicated anticoagulation, large thrombi > 60 mm where anticoagulation was not previously used, concomitant cardiac abnormalities that could be corrected in the same setting like presence of patent foramen ovale (PFO), and endocarditis with a surgical indication [6].

It was used as a first choice, as reported by Stavroulopoulos et al., in 17 patients and in 5 patients after failure of medical treatment. Three cases died postoperatively. Comparing survivors with nonsurvivors in the surgery group, age was the only significant difference, 36.5 ± 12.4 versus 52.7 ± 5.8 years, respectively, *P* = 0.049. To note, six patients were anticoagulated after the surgical extraction of the thrombus [1]. It should be borne in mind that the patient population with CRT has a significant likelihood of being poor surgical candidates given their comorbidities.

Anticoagulation has been used as a first line or as an adjunctive treatment following other interventions. It was successful in most cases and was usually used for 6 months. Comparing 37 cases who received anticoagulation to 23 surgical thrombectomies, there was no significant mortality difference, 16.2% and 13%, respectively, *P* = 1. The median value of thrombus size was 28 mm in both groups [1].

Percutaneous intravascular removal of CRT represents another treatment modality however due to risk of mechanical dislodgment, perforation, and the technical difficulties especially in dealing with large thrombi; its use is advocated when other treatment modalities are contraindicated [7].

The emergence of ultrasound assisted catheter directed thrombolysis (The EKOS EndoWave Infusion Catheter System (EKOS Corporation, Bothell, WA)) as a method of local thrombolytic delivery provides another possible treatment modality for CRT. The ultrasound waves accelerate the fibrinolytic process by enhancing catheter directed thrombolysis. This in turn reduces the treatment time and total thrombolytic dose resulting in less risk of bleeding [8]. To our knowledge, two prior cases were reported so far, both used in cases of right atrial thrombi that were not dialysis catheter associated, and both had concomitant PE. Nickel et al. reported a case of failed EKOS use as 48 hrs later there was a residual occlusive thrombus in the suprarenal IVC extending to the cavoatrial junction that was successfully retrieved using the Vortex Angiovac system [9] while Shammas et al. reported successful EKOS use with complete resolution of the thrombus 24 hours later as evidenced by echocardiogram [10]. We include a comparison between our case and prior reported cases of EKOS catheter use [Table 1].

In our case, the use of EKOS resulted resolution of the thrombus. Subsequently, systemic anticoagulation with warfarin was initiated and our patient was discharged home in stable condition. To our knowledge, this is the first reported case of EKOS used in the treatment of CRT without concomitant PE.

## 4. Conclusion

Catheter related thrombosis is a commonly encountered entity fraught with substantial risk for mortality. Determining the best treatment approach that guarantees complete resolution of the thrombus while avoiding fragmentation and ensuing pulmonary embolism poses a challenge for physicians. We believe that the decision should be individualized after carefully deliberating potential risks associated with surgical intervention, hemorrhage with systemic thrombolysis, technical difficulties with percutaneous retrieval, and the size of the thrombus. EKOS emerges as another modality with a relatively lower risk of hemorrhage compared to systemic thrombolysis; however, extreme caution and experience are required to avoid thrombus fragmentation during the catheter insertion.

## Supplementary Material

TEE video, Aortic valve short-axis view, showing the right atrial thrombus prolapsing into the right ventricle.

## Figures and Tables

**Table 1:** Comparison between three cases where EKOS was used for treatment of a right atrial thrombus.

Case	Shammas et al. [10]	Nickel et al. [9]	Our case
Age	69	62	75

Gender	Male	Female	Male

Risk factors for thrombosis	Limited activity (familial spastic paraparesis)	Metastatic melanoma IVC filter	Prostate cancer permacath

Thrombus size	2.8 × 2.3 cm	8.2 × 4.1 cm	8.4 × 0.5 cm

Attachment	Interatrial septum	A thin attachment to the cavoatrial junction in the region of IVC stent (large volume thrombosis extending from the femoral veins bilaterally, into the vena cava and proximal right atrium)	Along anterolateral Rt atrium

Mobility	Mobile	Mobile	Highly mobile

Prolapsing into RV	No	Yes	Yes

Presence of PE	Bilateral extensive PE with near occlusion of Rt main trunk and nonocclusive thrombus at left PA	Chronic PE with mildly enlarging emboli in the left main and right posterior segmental pulmonary arteries (status post-prior IVC filter placement)	No

Vitals on admission	SBP 80, HR 120, 98% 5-6-liter NC	N/A	95/60, 97, 97%

EKOS catheter	24 cm and 12 cm	N/A	12 cm/135

Site of EKOS catheter	(24) junction between RA and IVC into Rt main PA extending into the thrombus (12) through Rt CFV to left PA	N/A	Into the right atrium and the right ventricle

Thrombolytic type	tPA	Alteplase	Alteplase

Duration	12 hrs	24 hrs, then 48 hrs	10 hrs

Dose	24 mg total (1 mg/hr for 12 hours each total 24 mg)	48 mg total (1 mg/hr for 24 hours, then 0.5 mg/hr for 48 hours)	25 mg total, 5 mg bolus, then 2 mg/hr

EKOS access	Bilateral CFVs	Popliteal veins	Left subclavian vein

Outcomes	(i) Echo postprocedure immediately: Rt atrial thrombus not dislodged (ii) Echo 24 hours later: complete resolution of Rt atrial thrombus (iii) CTA: marked improvement of Rt PA thrombus and lt PA	(i) Venography: grade III lysis in the pelvic veins (99-100%), with residual occlusive thrombus in the suprarenal IVC, superior to the filter, extending to the cavoatrial junction (ii) After 48 hrs of reinitiated EKOS: persistent filling defect in the suprarenal IVC (iii) Venoplasty was done with extrusion of the cavoatrial filling defect into the right atrium. Managed with Angiovac	Resolution of the thrombus (continued on warfarin)

Other modalities used	24 hrs of heparin, then rivaroxaban	Heparin Stenting Endovascular retrieval using the Vortex Angiovac system (AngioDynamics, Marlborough, MA)	Heparin, then warfarin
